# Risk taking for potential losses but not gains increases with time of day

**DOI:** 10.1038/s41598-023-31738-x

**Published:** 2023-04-04

**Authors:** Rachel L. Bedder, Matilde M. Vaghi, Raymond J. Dolan, Robb B. Rutledge

**Affiliations:** 1 Max Planck UCL Centre for Computational Psychiatry and Ageing Research, University College London, London, UK; 2 Wellcome Centre for Human Neuroimaging, University College London, London, UK; 3 Department of Psychology, Yale University, New Haven, USA; 4 State Key Laboratory of Cognitive Neuroscience and Learning, IDG/McGovern Institute for Brain Research, Beijing Normal University, Beijing, China; 5 School of Psychology, University of East Anglia, Norwich, UK

**Keywords:** Psychology, Human behaviour

## Abstract

Humans exhibit distinct risk preferences when facing choices involving potential gains and losses. These preferences are believed to be subject to neuromodulatory influence, particularly from dopamine and serotonin. As neuromodulators manifest circadian rhythms, this suggests decision making under risk might be affected by time of day. Here, in a large subject sample collected using a smartphone application, we found that risky options with potential losses were increasingly chosen over the course of the day. We observed this result in both a within-subjects design (N = 2599) comparing risky options chosen earlier and later in the day in the same individuals, and in a between-subjects design (N = 26,720) showing our effect generalizes across ages and genders. Using computational modelling, we show this diurnal change in risk preference reflects a decrease in sensitivity to increasing losses, but no change was observed in the relative impacts of gains and losses on choice (i.e., loss aversion). Thus, our findings reveal a striking diurnal modulation in human decision making, a pattern with potential importance for real-life decisions that include voting, medical decisions, and financial investments.

## Introduction

Everyday decisions are driven by risk attitudes that vary across individuals^1–3^. Furthermore, risk preferences can differ when potential gains and losses are concerned. The same person that takes a cursory glance at a red walk signal before hurrying on their way (i.e., risk seeking in a loss frame) might always stick to an old favorite at an ice cream shop (i.e., risk aversion in a gain frame). The reflection effect refers to a reversal of preferences when shifting between gain and loss magnitudes. This effect is a central feature within prospect theory^4^, and is repeatedly observed at the population level^5,6^. It is also robust across multiple demographics including, for the most part, a sample of 4098 participants spanning 19 countries and 13 different languages^7^. This effect is also observed in non-human primates^8^. Behaviorally the reflection effect manifests as risk aversion for gains (i.e., smaller certain reward are preferred over larger risky rewards) and risk seeking for losses (i.e., larger risky losses are preferred over smaller certain losses^4^.

Biological accounts link risk-taking behavior to underlying neural circuitry^9^, and to processes that change across the lifespan (reviewed in development:^10^, and in ageing^11^). These biological changes are consistent with epidemiological evidence that shows that adolescents engage in greater risky behaviors^12^, which is often attributed to developmental changes in dopaminergic reward circuitry^13,14^. However, the majority of such studies focus on situations in which every risky option includes a potential gain, without considering decisions that involve only potential losses. Many studies implicitly assume that risk taking for gains and losses is governed, at least partially, by shared processes^12^. However, natural aging is associated with a decline in the integrity of the dopamine system (e.g.,^15^) and this has been linked to a parallel decline in risk taking for potential gains but not potential losses^16^. In a broader sample between the ages of 12–90, risk attitudes for gains did not relate to risk attitudes for losses in the same individuals^6^. The overall pattern of findings from these behavioral studies suggests that risk taking for gains and losses might be controlled by distinct processes with at least partially dissociable biological underpinnings.

Similarly, computational modelling in studies of risky decision making often feature a single risk aversion parameter and a loss aversion parameter. Loss aversion is assumed to govern the relative impact of potential gain and losses on choice (i.e., ‘losses loom larger than gains’^4,17^), which could influence risk taking related to both potential gains and losses (reviewed in^18^). Notably, this parameter can be difficult to identify specifically in paradigms with a large range of probabilities using the cumulative prospect theory model e.g.^19,20^,that is closely related to the model we employ here for simpler paradigms. Furthermore, many studies do not consider situations in which only potential losses are involved. In line with dual-process models of decision making, dopamine is associated more with reward-related behaviors^21^, while serotonin is associated more with loss-related behaviors^22^. An environmental asymmetry in rewarding and punishing stimuli has been proposed as providing an adaptive account for dissociable decision processes that could vary independently^23^. Many neurobiological processes are known to change over the course of the day (e.g., hormones^24^; digestion^25^), and this includes regular fluctuations in neuromodulators^26–28^. There are circadian changes in risk taking in animals, although the mechanistic basis of those changes is not well understood. Circadian changes in risk taking are observed in male dark-eyed juncos deciding whether to sing or forage^29^, and increased risk taking during foraging is observed later in the day in sparrows^30^. Some risk-taking behaviors have been shown to change according to time of day in humans, including decreased sensitivity to negative feedback in the afternoon compared to the morning^31^, and greater engagement with reward-driven behaviors in the evening (e.g., consuming higher calorie food^32^). Thus, we hypothesize that time of day influences risk taking in humans, and investigate whether effects differ for potential gains or losses.

We tested risk attitudes for gains and losses in humans as a function of time of day. To this end, we measured propensity to take different types of risks at different time of the day in a within-subject design, availing a sample of 2599 participants who played a gamified risky decision making task more than once on a smartphone platform (*The Great Brain Experiment*)*.* We implemented computational models based on prospect theory, to test whether risk taking for gains and losses show differential diurnal patterns that might reflect a differential mechanistic basis. To test the generalizability of our result, we looked at the first play of more than 25,000 participants and split for demographics including age and gender.

## Methods

### Participants

We included the data from 26,720 participants from the UK and USA aged 18–69 (18,106 participants between ages 18–39; 13,054 female participants; 18,977 participants from the UK) who completed a risky decision task between March 1, 2013 and September 30, 2014 on the gamified cognitive task platform *The Great Brain Experiment.* Participants played the task at whatever time they liked with no prompting. Results from a smaller subset of this data set have been previously published elsewhere^16^. The analyses presented here were exploratory and not pre-registered. Gender, age, and location were defined as participants self-selecting their demographics when they downloaded the app. Participants selected their age from the given age brackets of: 18–24, 25–29, 30–39, 40–49, 50–59, 60–69 and 70+. Participants selected the country they were based in from a list. All participants gave informed consent within the smartphone platform. The Research Ethics Committee of University College London approved this study and adhered to the tenets of the Declaration of Helsinki.

### Smartphone-based experiment

**Figure 1:**
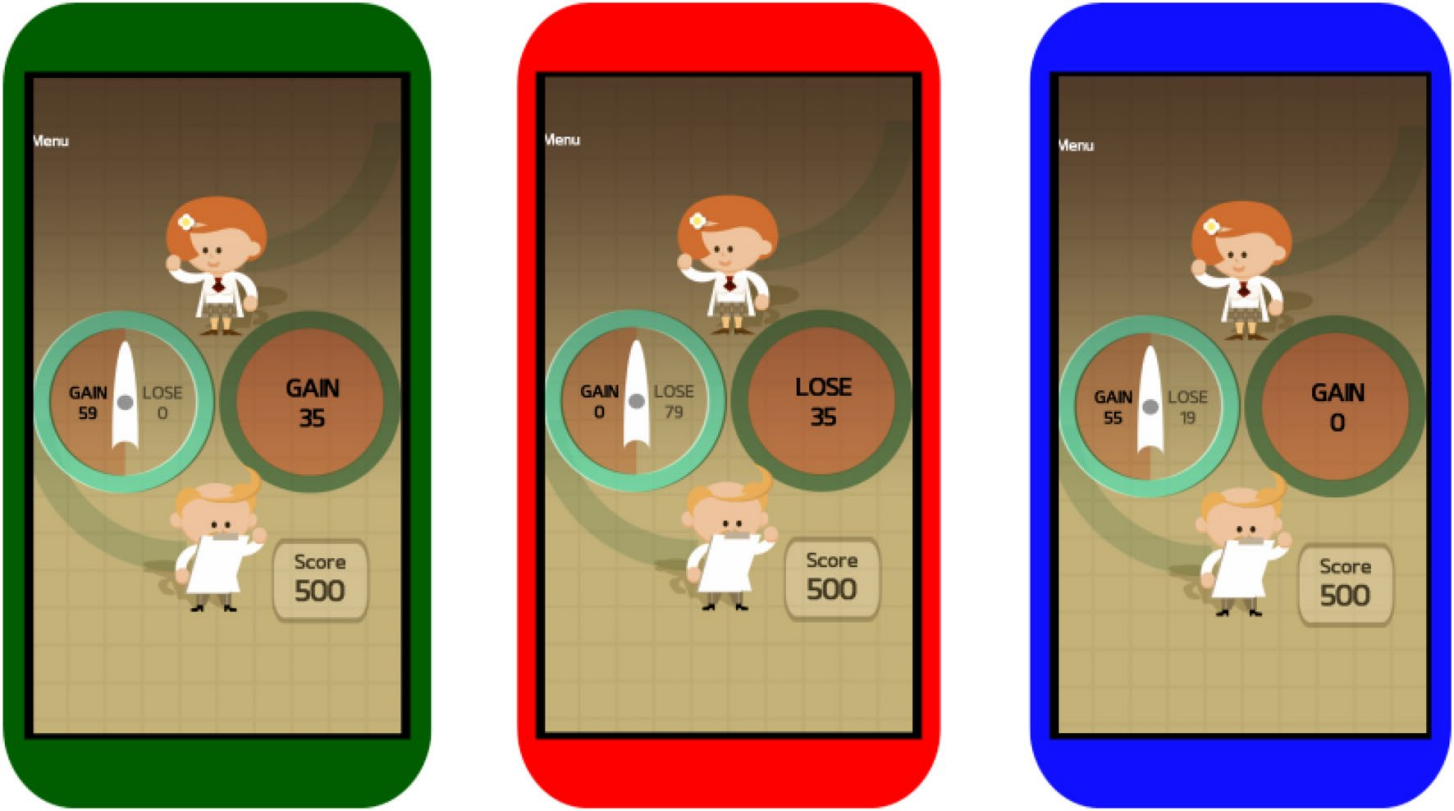
Task Design. Gain trials (green outline) had potential gains and no potential losses. In an example gain trial, a participant chose between a risky option (here, 50% probability of 59 points) and a safe option (here, 100% probability of 35 points). Loss trials (red outline) had potential losses and no potential gains. Mixed trials (blue outline) had both potential gains and potential losses with a safe option that was always worth 0 points. Therefore, on any trial participants could opt for a risky choice. The experimental design enables the role of a gain or loss magnitudes in propensity towards risk to be disambiguated. The participants total score, starting at 500 points, was added or taken away from throughout the game as they win and lose points on each risky choice. The green, red, and blue outlines were added to the figure for descriptive purposes, each trial had the same appearence to the participants.

The task involved 30 trials where participants chose between safe and risky options. Participants also rated their happiness 12 times and the results of those analyses are reported elsewhere^33,34^. The majority of participants took between 3 and 5 minutes to complete each game. Participants began with 500 points. When each game was finished the participants were told the score they achieved, what percentage of all other plays from all players it was higher than, and their all-time high score. On each trial participants chose between a safe option (where the points were guaranteed if they chose the option) or a risky option where they had a 50% chance of two potential outcomes. The risky choice was represented on a spinner where an arrow moved around until it landed on either of the two potential outcomes (Fig. 1). Where the safe option was chosen the outcome was resolved immediately. There was no time constraint on making a decision on each trial.

Each play contained three types of trials: (1) 11 gain trials, where they could choose between a certain gain and a gamble with a greater potential gain or a zero; (2) 11 loss trials, where they chose between a certain loss and a gamble with a greater potential loss or a zero; and (3) 8 mixed trials where they chose between a certain option of zero or a gamble with a potential loss and a potential gain. Two different design matrices were used (ratio and uncorrelated, for more details see the Supplementary Methods). Each trial began with the instruction ‘Spin the Spinner or Play it Safe!’ and the green borders on the circles which contained each of the two options alternated between light and dark green every few seconds to indicate to the participants that either option can be selected by tapping the screen (Fig. 1).

### Data analysis

All data from the smartphone platform was time stamped with Greenwich mean time (GMT). Only country-level location data was available for all participants. Thus 6.5 h was subtracted from timestamps for data collected in the USA to correct for the time difference for the average participant. 2599 participants completed the game at least twice, allowing a within-subject analyses. Eligibility for this subset was defined as having completed at least two game plays between 8 am and 10 pm on different days that were both either the ratio or uncorrelated designs. These time windows were utilized in previous studies^35,36^. 26,720 participants completed the game at least once, allowing for a between-subjects analysis comparing age groups, genders, and different design matrices in their first play. Analysis of first plays only in this extended sample avoids some potential issues with self-selection based on interest in completing a risk-taking task at particular times of day, because the smartphone app features multiple games related to different aspects of cognition and participants could not predict the nature of the game before selecting to play. Participants do not know before they start the game that it involves risky decisions.

We report Pearson correlation coefficients for effect sizes of relationships between task measures and time of day of the game’s completion. Additionally, we computed the difference between pairs of effect sizes between time of day and parameters of a computational model, to test if time of day had different effects on each parameter. All p values were computed based on permutation tests using 10,000 random shuffles of the time of play to determine null distributions (MATLAB, Version 2018a). We also included Bayes Factor tests (JASP, Version 0.14.1). When the Bayes Factors were reported in the main body of the text, we report BF_01_ when the p value exceeds 0.05, and BF_10_ when the p value is less than 0.05. A BF of more than 1 indicates support for the null (BF_01_), or alternative hypothesis (BF_10_). A BF of less than 3 offers mild evidence for the hypothesis, a BF between 3 and 10 offers moderate evidence, and values exceeding 10 offer strong evidence. When Bayes Factors are reported in a table, they are all given as BF_01_ for ease of comparison. When the Bayes Factor is reported as ∞ this means the magnitude exceeds what is computable with the software. We computed these analyses separately for both genders, and for older and younger players, and for USA and UK participants. We fitted choices to individual game plays with a prospect theory model using maximum likelihood, where the subjective utility of the gamble and certain options were determined by a separate power function for the gain and loss values (loss sensitivity: α_loss_, and gain sensitivity: α_gain_) and a multiplier for any loss values (loss aversion: λ).

$$U_{gamble} = 0.5\left( {V_{gain} } \right)^{{a_{gain} }} - 0.5\lambda \left( {V_{loss} } \right)^{{a_{loss} }}$$

$$U_{certain} = \left( {V_{certain} } \right)^{{a_{gain} }} \quad {\text{if}}\;V_{certain} \ge 0$$

$$U_{certain} = - \lambda \left( { - V_{certain} } \right)^{{a_{loss} }} \quad {\text{if}}\;V_{certain} < 0$$

Choice probabilities were then determined by a softmax rule which converts the difference in subjective utilities to the probability of gambling. The softmax includes an inverse temperature parameter ($\upmu$) which quantifies choice stochasticity.

$$P_{gamble} = \frac{1}{{1 + {\text{e}}^{{ - {\upmu }\left( {U_{gamble} - U_{certain} } \right)}} }}$$

## Results

On each choice trial, participants were presented with choices between risky and safe options and had to activate a spinner to make a risky choice. To detect the presence of diurnal variations in risk taking, we examined how preference for risk taking in gain, loss, and mixed trials (Fig. 1) changed according to the time of day that the task was completed. Including trials which contain gains and losses separately allowing us to investigate the roles of each frame on risk throughout the day, rather than just attitudes to risk generally. We used a within-subjects analysis in 2599 participants who had completed plays on two different days. To test how generalizable our results were to multiple demographics such as age and gender, we examined first plays alone using an extended sample of 26,720 participants, who played at different times of day. The median time the task was completed in the extended sample was 18:48 with 70% of plays between 08:00 and 22:00.

### Within-subject analysis

To test for the presence of circadian effects within individuals, we used the first two plays from players (N = 2599; female = 1327; 18–39 = 1507) who completed plays on two different days between 08:00 and 22:00 (according to GMT). If risk taking increases throughout the day, then small differences in the time of day for the two plays should be associated with little difference in behavior, but large differences should be associated with greater differences in risk taking. For each subject, we computed the difference in the time of day between the later and the earlier play, where a larger difference indicates the later play being collected further on in the day compared to the earlier play. The mean time of day difference between each play used in the analysis was 4.3 h (SD, 3.4 h). 52% of subjects (N = 1350) had the game played at the earlier time of day on a later calendar day. Thus, the earlier and later plays are balanced for whether they were performed first or second. Thus, prior experience or learning would be unlikely to account for the changes we observed with time of day.

**Figure 2:**
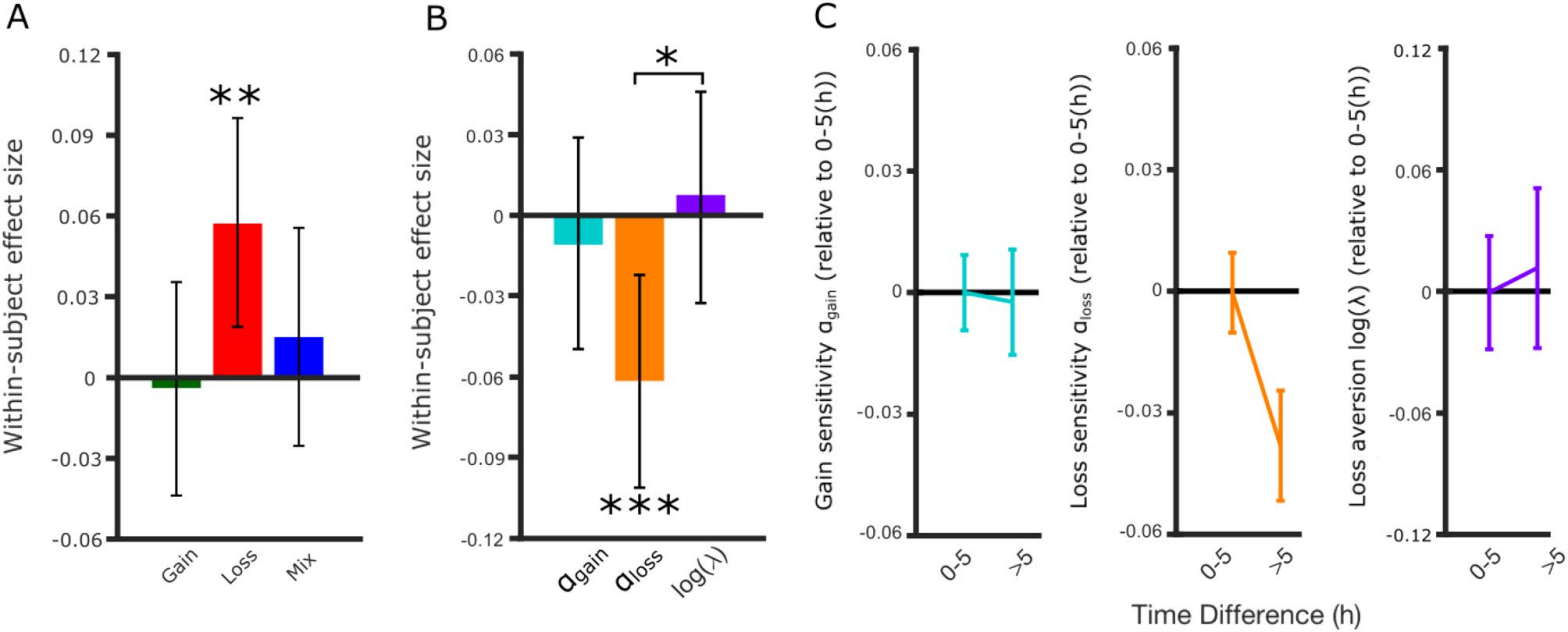
Loss sensitivity decreases with time of day in individuals. (**A**) We identified individuals (N = 2599) who completed the risky decision task on two different days between 08:00 and 22:00. Risk taking in loss trials increased with time of day within individuals. (**B**) Loss sensitivity (α_loss_), but not gain sensitivity (α_gain_) or loss aversion (log(λ)), decreased with time of day in this within-subject sample. Error bars represent bootstrapped 95% confidence intervals [*p < 0.05, **p < 0.01, ***p < 0.005]. (**C**) Loss sensitivity was lower for later plays in within-subject analysis. Error bars represent bootstrapped 95% confidence intervals. Data binned on differences less (N = 1,643) or more than 5 h (N = 956) are shown for illustration purposes. [*p < 0.05, **p < 0.01, ***p < 0.005].

First, we found that in gain trials (i.e., those with no potential losses) there was no significant relationship between the difference in proportion of risky choices made (for the later play minus the earlier play) and the difference in time of day (Pearson’s r =  − 0.0038, *p* = 0.84, BF_01_ = 39.93). In contrast, in loss trials (i.e., those with no potential gains), we observed a positive correlation between time of day difference and difference in risk taking between the two plays (r = 0.057, *p* = 0.0038, BF_10_ = 1.58). We did not observe an effect in mixed trials (r = 0.015, *p* = 0.45, BF_01_ = 30.40) (Fig. 2A). Hence, risk taking for potential losses, but not potential gains, increases with time of day, with people on average choosing more risky options with potential losses later compared to earlier in the day. Because participants could opt for a risky choice in any of the three types of trials, these results suggest that time of day selectively affects loss-related behavior (i.e., loss and mixed prospect choices) and does not affect risk taking in gain trials (i.e., gain choices).

### Computational modelling using prospect theory

Choice behavior in risky decision tasks is well described using a parametric computational model based on prospect theory^4,37^. Standard decision models include parameters for risk aversion (α), loss aversion (λ), and choice stochasticity (inverse temperature, μ). This model can be extended to allow risk aversion to vary for gains and losses separately (referred to as gain sensitivity, α_gain_ and loss sensitivity, α_loss_). Adopting a model-based approach to our behavioral data allows us to delineate between alternative mechanisms which could explain the time of day effect on risk taking for losses but not gains, asking whether time of day affects a single model parameter. An increase in risk taking for trials that include losses could be explained by a decreased loss sensitivity (α_loss_), reducing the difference in expected utility between potential losses associated with safe and risky options. One effect of a decrease in loss sensitivity is to render the safe option subjectively less attractive relative to the risky option. Loss aversion is perhaps the best known of the phenomena described in prospect theory. Increased risk taking for losses could also relate to a decrease in loss aversion (λ), a parameter that captures the relative weighting in choice of potential losses and equivalent gains.

We fit a model with a single risk aversion parameter (Single Alpha Model) and a second model with separate risk parameters for gains and losses (Dual Alpha Model) for each play to each participant separately. The Dual Alpha Model (pseudo-r^2^ = 0.40 ± 0.24, mean ± SD) fit the data better than Single Alpha Model (pseudo-r^2^ = 0.31 ± 0.22) and was preferred according to Bayesian model comparison which penalizes for model complexity (Table 1).

**Table 1:** Model comparison for within-subject data set.

Model	Parameters per subject	Mean r^2^	Median r^2^	Model BIC	BIC-BIC_dual_
Single alpha	3	0.31	0.27	201,608	1705
Dual alpha	4	0.40	0.36	199,903	0

BIC measures are summed across the within-subject dataset (N = 2599). Both models included choice stochasticity (inverse temperature) and loss aversion parameters. The final column is the difference between the model BIC and BIC for the Dual Alpha model.

Using the preferred Dual Alpha Model, we compared the estimate of each parameter between the later and earlier play. As expected, gain sensitivity (α_gain_, 1.02 ± 0.31, mean ± SD), was not associated with time of day (r =  − 0.011, *p* = 0.59, BF_01_ = 34.94). Instead, loss sensitivity (α_loss_, 0.82 ± 0.34) was correlated with time of day (r =  − 0.061, *p* = 0.0019, BF_10_ = 3.01), where decreased α_loss_ is consistent with the observed increase in risk taking in loss trials with time of day (Fig. 2B,C). If loss aversion decreased with time of day, this could partially explain increased risk taking for losses. However, we did not find a relationship between loss aversion log(λ) (0.59 ± 0.93) and time of day difference (r = 0.0075, *p* = 0.70, BF_01_ = 37.79) (Fig. 2B,C). We found that the strength of the decline in loss sensitivity over the course of the day was significantly greater than any decline in loss aversion (*p* = 0.015). We also found no relationship between time of day and choice stochasticity µ (0.96 ± 3.25, r =  − 0.020, *p* = 0.309, BF_01_ = 24.07), arguing against an explanation for our results in terms of changes in choice randomness. We report the effects of time of day on the parameters in the Single Alpha Model in the Supplementary Results (S3).

In more complex versions of prospect theory^38^, it has been shown that it is often difficult to identify the independent contributions of risk aversion and loss aversion. In order to test whether the time of day effect could be attributed to changes in loss aversion, we simultaneously fit the two plays (60 trials) from the within-subjects group using the winning Dual Alpha Model first allowing for only λ to vary separately for each fit (Split Lambda Model) and secondly allowing for only α_loss_ to vary separately for each fit (Split Alpha Loss Model). To assess whether the observed time of day effects could be accounted for in models that only allowed variation for either α_loss_ or λ but not both, we simulated choices using the parameters estimated from each new model fit and tested whether simulated rates of risk taking correlated with the difference in time of day between the two plays. If loss aversion can explain the observed effect of time of day on risk taking in loss trials, we would expect simulated choices from the Split Lambda Model to show greater risk taking in loss trials with time of day. For the Split Lambda model, neither simulated choices in mixed trials or loss trials correlated with time of day (mixed: r = 0.002, *p* = 0.92, BF_01_ = 40.48; loss: r =  − 0.0076, *p* = 0.70, BF_01_ = 37.78). As expected, the Split Alpha Loss model showed a significant increase in risk taking in loss trials with time of day (loss: r = 0.047, *p* = 0.018, BF_10_ = 0.044)_,_ but not with mixed trials: r = 0.019, *p* = 0.35, BF_01_ = 26.08). These simulated results were consistent with the model-free results for the within-subjects data, except the Bayes Factor tests (BF_10_) did not support the alternative hypothesis for the loss trials. Thus, simulations with new models that only allow a single parameter to be different across plays show that λ alone is unable to account for the pattern of model-free results that we observed in the within-subjects data. In contrast, α_loss_ alone is able to account for the observed pattern of model-free results.

### Between-Subjects analysis

For the within-subjects analysis we used only participants who had played the game twice and on different days. To address whether our results generalize to different age groups, genders, and other subgroups of data (Table 2) we took advantage of a larger sample of participants (N = 26,720, UK = 18,977; USA = 7743 including those from the within-subjects analysis). We used the first completed play from each participant from all 24 h of the day. For all analyses we considered the day to begin at 06:00.

**Table 2:** Effect sizes for time of day on risk taking for each demographic split.

Data	Sample size	Gain trials		Loss trials		Mixed trials	
		Effect size	*p* Value BF_01_	Effect size	*p* Value BF_01_	Effect size	*p* Value BF_01_
All	26,720	0.0018	0.77 124.94	0.037	< 0.0001 1.52 × 10^−6^	0.027	< 0.0001 0.009
UK	18,977	− 0.00035	0.96 109.79	0.040	< 0.0001 2.85 × 10^−5^	0.029	< 0.0001 0.044
US	7,743	0.011	0.34 44.00	0.027	0.016 4.12	0.021	0.066 12.80
Male	13,666	− 0.0038	0.65 84.38	0.032	0.0002 0.098	0.011	0.20 40.54
Female	13,054	0.0052	0.56 76.50	0.040	< 0.0001 0.0030	0.038	< 0.0001 0.0090
Younger	18,106	− 0.0068	0.39 70.54	0.039	< 0.0001 1.56 × 10^−4^	0.025	0.0006 0.44
Older	8,614	0.0045	0.68 67.83	0.036	0.0014 0.31	0.037	0.0007 0.23
Ratio	7,750	− 0.0026	0.82 68.48	0.046	< 0.0001 0.017	0.029	0.010 2.71
Uncorrelated	18,970	0.0073	0.31 66.52	0.028	< 0.0001 0.075	0.022	0.0031 1.04

Effect sizes (Pearson’s r) for each demographic and experimental split of the data (country, gender, age, ratio or uncorrelated task designs). Demographic pairs (where total N = 26,720) are every pair of rows after ‘All’. All Bayes Factor tests test for evidence for the null hypothesis (BF_01_). BF_01_ of > 1 indicate support for the null hypothesis. We report the mean, standard deviations and medians for the full sample, and male and female demographic splits in the Supplementary Results (S1). We also report the effect sizes for the proportion of choices with the highest expected value and total points scored for each trial type in the Supplementary Results (S2).

**Figure 3:**
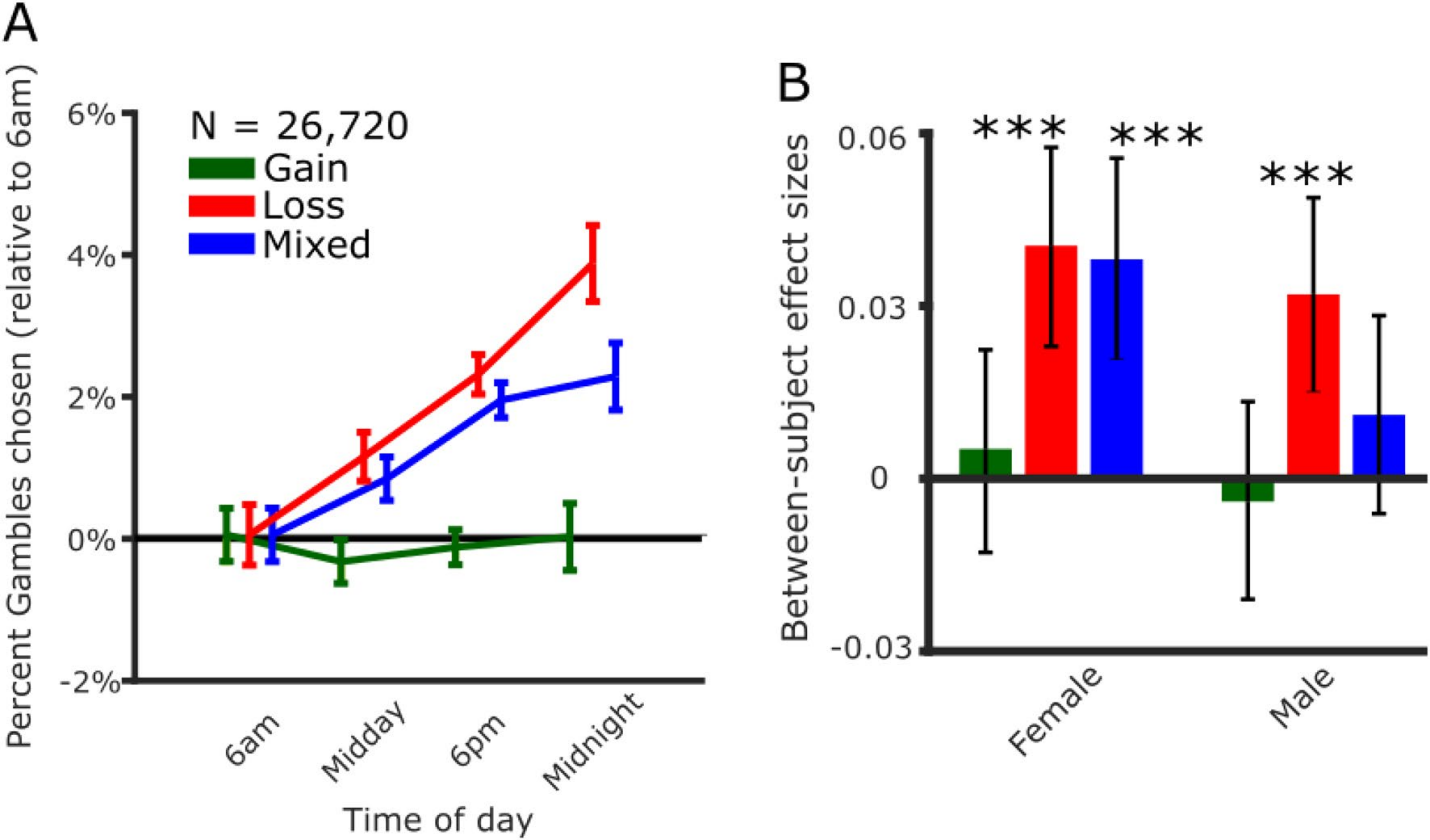
Risk taking for potential losses increases with time of day. (**A**) Risk taking in choice trials with potential losses increased with time of day (N = 26,720, first plays only). This was true in loss trials featuring risky options with equal probabilities of zero or a potential loss, and in mixed trials featuring risky options with equal probabilities of potential gains and losses. The frequency of choosing risky options with only potential gains was unaffected by time of day. Each time bin includes data collected in the interval starting at the time indicated (i.e., the 6am bin includes all data collected from 06:00 until 11:59). Error bars represent the SEM. (**B**) Risk taking in loss trials increased with time of day in both female (N = 13,054) and male participants (N = 13,666). Risk taking in mixed trials increased with time of day in females but not males. Error bars represent bootstrapped 95% confidence intervals [*p < 0.05, **p < 0.01, ***p < 0.005].

We were able to confirm our results from the within-subjects sample, where the number of risky choices in loss trials and time of day showed a positive correlation (r = 0.037, *p* < 0.0001, BF_10_ = 655,526.74). We also found a positive correlation between risky choices in mixed trials, which feature both potential gains and losses (r = 0.027, *p* < 0.0001, BF_10_ = 106.61) (Fig. 3) which we did not observe in the within-subjects sample. Consistent with the within-subjects sample, we observed no significant relationship in gain trials (r = 0.0018, *p* = 0.77, BF_01_ = 124.94). Time of day effects for loss and mixed trials did not differ significantly from each other (*p* = 0.068), but effect sizes for mixed and loss trials were both significantly greater than for gain trials (both *p* < 0.0001).

We next looked to see if this effect was present in multiple subsamples of the first plays. This diurnal effect on risk taking was robust to gender in loss trials (female: r = 0.040, *p* < 0.0001, BF_10_ = 388.26; male: r = 0.032, *p* = 0.0002, BF_10_ = 10.24). In mixed trials the effect was statistically significant for female (r = 0.038, *p* < 0.0001, BF_10_ = 116.93) but not male participants (r = 0.011, *p* = 0.20, BF_01_ = 40.54). Positive effect sizes were seen for younger and older age groups for loss trials (18–39: r = 0.039, *p* < 0.0001, BF_10_ = 6409.96; 40+: r = 0.036, *p* = 0.0014, BF_10_ = 3.28, and mixed trials (18–39: r = 0.025, *p* = 0.0006, BF_10_ = 2.3; 40+: r = 0.037, *p* = 0.0007, BF_10_ = 4.33). The direction and significance of these effects was consistent when the sample was split for two different trial design matrices (i.e., ratio and uncorrelated) (Table 2). Overall, increased risk taking in loss but not gain frames was a highly consistent result (Table 2). Although increased risk taking in mixed trials (most closely associated with loss aversion in the literature) was also robust to age and task design, the effect was present in female participants, but no significant effect was found in male participants.

To further assess the robustness of the results in light of the lack of information regarding the precise time zone, we jittered the time of day for the USA sample using 0.5 and 1.5 plus or minus as a conservative test (i.e., the time zone cannot be off by more than 1.5 h). We repeated the test 100 times and found that in the USA sample time of day was consistently associated with increasing risk taking for losses (*p* < 0.05 in 95% of samples), but not gains (*p* < 0.05 in 2% of samples). Since the within-subjects analyses relate to the difference in time between two plays (i.e., a single player on different days), these results would be unaffected by the knowledge of the exact time zone of our USA participants.

### Computational modelling in the between-subjects sample

**Figure 4:**
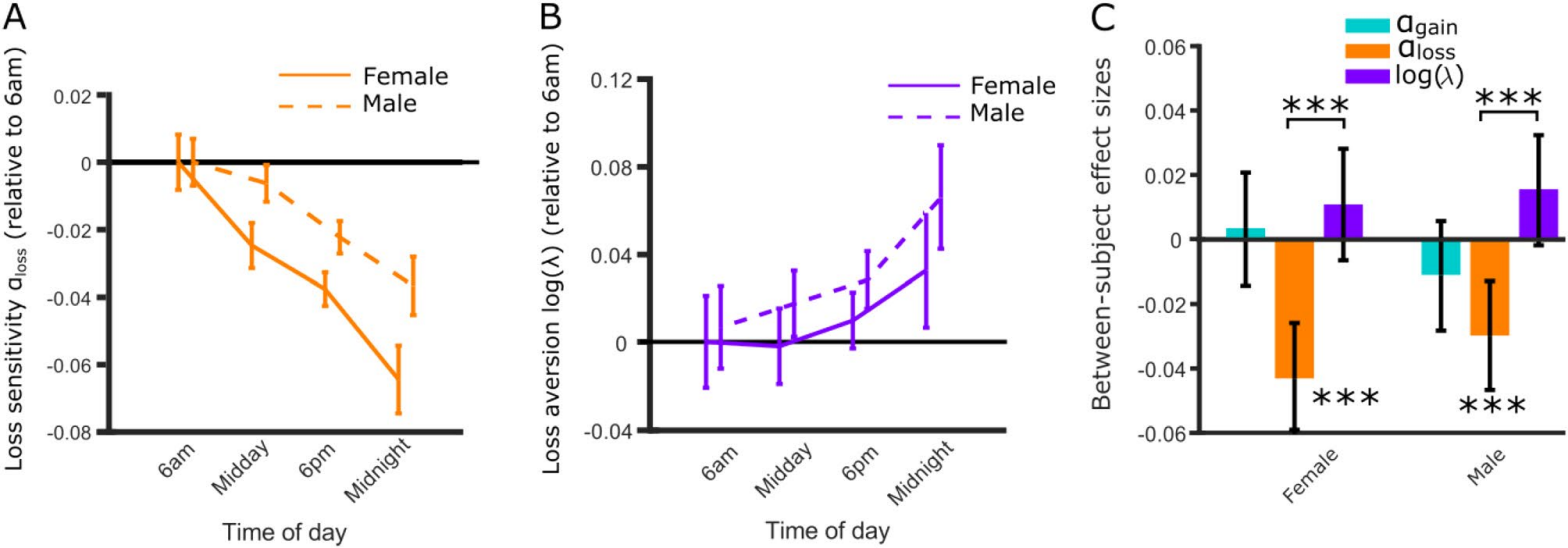
Loss sensitivity and not loss aversion decreases with time of day. (**A**) Loss sensitivity parameters (α_loss_) decreased with time of day in both females and males, consistent with increased risk taking in trials with potential losses. Error bars represent the SEM. (**B**) Increased risk taking for losses could be partially explained by decreased loss aversion. However, loss aversion parameters (log(λ)) did not decrease with time of day in both samples. Error bars represent SEM. (**C**) Loss sensitivity decreased more than loss aversion in both females and males. Error bars represent bootstrapped 95% confidence intervals [*p < 0.05, **p < 0.01, ***p < 0.005].

Using the Dual Alpha Model (also preferred by model comparison when using the full sample, Table 3) we examined each parameter in relation to the time of day the game was played. As expected, time of day was correlated with α_loss_ (r =  − 0.039, *p* < 0.0001, BF_10_ = 3.12 × 10^+6^ , Fig. 4A). This result was robust to gender (Fig. 4C), age, and task design (all r <  − 0.029, *p* < 0.001 BF_10_ > 10), in line with the model-free analyses. We also observed a modest increase in loss aversion log(λ) (r = 0.013, *p* = 0.036, Fig. 4B). However, the data does not provide evidence for the alternative hypothesis despite the large sample size with a BF_10_ of 0.068. Loss sensitivity α_loss_ decreased significantly more with time of day than loss aversion log(λ) in both female (*p* < 0.0001) and male participants (*p* = 0.0007). α_gain_ (1.02 ± 0.30, mean ± SD) was not associated with time of day (r =  − 0.0042, *p* = 0.50, BF_01_ = 103.49) nor was choice stochasticity μ (0.74 ± 2.76; r =  − 0.0071, *p* = 0.24, BF_01_ = 66.84).

**Table 3:** Model Comparison for individual plays.

Model	Parameters per subject	Mean r^2^	Median r^2^	Model BIC	BIC-BIC_dual_
Single alpha	3	0.29	0.25	1,061,299	1481
Dual alpha	4	0.37	0.34	1,059,817	0

Bayesian information criterion (BIC) measures are summed for fits for each participant’s first play (N = 26,720). Both models included choice stochasticity (inverse temperature) and loss aversion parameters. The final column is the difference between the model BIC and BIC for the Dual Alpha model.

To test whether the model parameters estimated for each participant were sufficient to generate the same time of day effect on risk taking, we sampled a new set of 30 trials for each participant (using the same design, ratio or uncorrelated, as they originally played) and simulated their choices on these new trials using their original fit parameter estimates. We confirmed that the expected time of day effect on the number of risky choices taken in each trial type was observed for simulated loss trials (r = 0.037, *p* < 0.0001, BF_10_ = 579,890.049), mixed trials (r = 0.027, *p* < 0.0001, BF_10_ = 153.11), but not gain trials (r =  − 0.0044, *p* = 0.47, BF_01_ = 100.73). Thus, the fits from our computational model are sufficient to show the selective effect we observed of time of day on risk taking for potential losses but not gains.

Many models suffer from inter-correlated parameters (including some versions of prospect theory; see^20^), and as a result, parameters cannot always be reliably estimated independently. We used a recovery analysis to test whether we could recover the differences in model parameters that were used to generate the data^38^. We fit our model to simulated choices to test whether we could recover the original parameter estimates. Recoverability was high for α_loss_ (r = 0.95, *p* < 0.0001, BF_10_ = ∞), log(λ) (r = 0.70, *p* < 0.0001, BF_10_ = ∞), and α_gain_ (r = 0.94, *p* < 0.0001, BF_10_ = ∞).

As a further test of whether 30 trials are sufficient to generate the time of day effect in data simulated from our model, we also confirmed the time of day effect on the recovered parameter estimates for α_loss_ (r =  − 0.033, *p* < 0.0001, BF_10_ = 23,951.08), but not α_gain_ (r =  − 0.0060, *p* = 0.33, BF_01_ = 80.46). The effect was significant for log(λ) (r = 0.019, *p* = 0.0027, BF_10_ = 0.78), but the Bayes Factor test did not show support for the alternative hypothesis.

## Discussion

We show that time of day constrains attitudes towards risk, informing a theoretic account of human behavior. Time of day, reflecting circadian rhythms, influences risk attitudes with respect to potential losses but with no impact on risk taking observed for situations with only potential rewards. Using computational modelling, we show that changes in risk taking for losses are explained by a reduced sensitivity to increasing losses over the day, which affects both loss and mixed prospect trials. The results are consistent with an account for risk taking with distinct processes related to risk taking for potential gains and losses. Humans make different decisions later in the day, and these differences depend on the valence of risky options.

In terms of computational processes, a model with separate value sensitivity parameters for potential gains and losses explained the data better than a model with a single risk aversion parameter. This was true in both within- and between-subject datasets, consistent with a time of day effect on one risk aversion parameter but not the other. For both model-free and model-based analyses, we see consistent results between the within-subjects and between-subjects data set, where the difference in the time of day the game was played correlated with the difference in risk taking for losses and loss sensitivity in the computational model. It is important to note that some studies have shown that estimating parameters for both loss aversion and risk aversion for gains and losses separately has poor parameter recovery for some task designs e.g.^19^. Furthermore, tasks with limited sets of probabilities can conflate estimates of risk aversion and loss aversion^39^. In order to assess whether shared variance between these parameters may disguise time of day changes in loss aversion, we tested whether change in a loss aversion parameter alone were sufficient to explain differences in behavior between the two plays for the within-subjects sample. We demonstrated that the difference in these parameter estimates when all other parameters were constrained to be the same in both plays did not correlate with time of day. Furthermore, this model could not account for simulated increases in risking taking in loss or mixed trials as a function of time of day.

Another possible limitation of our between-subjects analysis is participants self-selecting into the time of day they played the game. However, because these analyses featured only individuals completing the task for the first time, they were naïve to the nature of the task. Because the smartphone app included multiple tasks related to different aspects of cognition, participants would not have known before starting the task that it involved risky decisions and so could not have selected to play due to diurnal impact on preferences for certain kinds of game mechanics. Furthermore, the fact that in all analyses the effect was seen for losses but not for gain suggests that the effect was not driven by idiosyncrasies of the game itself (e.g., the spinner animation which is present for all trial types).

While we observe the effect in a within-subjects analysis and in multiple demographic groups, we acknowledge the effect sizes for these analyses are small. Smartphone and online data collection have been very successful in replicating many longer lab experiments, but have typically recorded a reduction in the observed effect sizes^40,41^. Such reductions in effect sizes may be expected due to inherent noise in this method, due to the small number of trials per participant and the real world outside of a laboratory setting. However, smartphone methods also benefit from being able to more easily sample outside standard young and educated laboratory demographics^42,43^ and results may be more likely to generalize to other contexts because participants have not had substantial interaction with and training from an experimenter. Recent research has demonstrated that tasks with similar designs without explicit incentives show similar choice behavior as to when they are financially incentivised^44^.

Gamified cognitive tasks in smartphones are well placed to uncover previously undetected temporal effects which can contribute to a more holistic account of day-to-day decision making. Typically, participants are not prompted as to when they should engage in tasks. While this can create selection effects when exploring the influence of circadian rhythms on behavior, particularly in between-subjects designs, we mitigate this issue by demonstrating consistent results in both between- and within-subjects analyses. In within-subjects analysis each subject acts as their own control, and thus if a participant with an increased tendency to gamble for losses was also most likely to complete the task in the evening, then playing twice in the evening would have little influence on the within-subjects time-of-day effect. Furthermore, the nature of our research question benefits from participants playing at unsociable hours of the day, where laboratory visits may be less welcome, creating a bias towards certain demographic groups being less willing or unable to attend visits early or late in the day for a lab-based experiment. In any such study, consistent patterns across between- and within-subjects analyses provides evidence that any consistent effects are robust to different self-selection effects.

Many neuromodulator processes are influenced by circadian rhythms, including dopamine, serotonin, noradrenaline and cortisol^26,27,45,46^. There is also evidence for a relationship with decision making for both cortisol (e.g., increases risk aversion for gains^47^), noradrenaline (e.g. reduces discrimination between loss amounts when the probability of winning was low^48^). Dopamine and serotonin are widely believed to play a role in decision making that relates primarily to gains and losses, respectively^23,49^. Selective Serotonin Reuptake Inhibitors (SSRIs) are associated with reduced attention to negatively valenced stimuli^50^. Depletion of tryptophan (the precursor of serotonin) reduces risk taking for losses^51^. Although dopamine is often associated with risk taking for reward^21,52^, there is also evidence that dopamine represents the value of loss-related stimuli^53^, and sensitivity to losses is associated with low dopamine^54^. Increased reward seeking when pre-stimulus dopaminergic midbrain BOLD activity is low and phasic responses to potential rewards are elevated provides a potential mechanism linking the dynamic range of dopamine and reward seeking^55^. If another neurobiological process related to aversiveness (i.e., serotonin) has a dynamic range that decreases with time of day, this could provide a potential mechanistic explanation for reduced risk aversion for losses. Future studies could measure tonic and phasic dopamine and serotonin throughout the day in relation to aversive stimuli to test whether either neuromodulator accounts for changes in risk taking with time of day.

An alternative account is that making decisions about losses increases task attention which may interact with changes in wakefulness through the day^56^. Previous diurnal patterns of behavior have been observed in the literature when taking into account a participant’s chronotype e.g.,^57,58^. For example, when participants performed a risk taking task with prospective gains at the time of day mismatched to their chronotype (i.e., whether they were a ‘morning type’ or ‘evening type’), risk taking increased but there were no changes in choice consistency^59^. Such differences are attributable to changes in wakefulness and reduced inhibitory executive function^35,60^. Here, we saw no change in choice stochasticity for simple value-based choice despite the large sample size. Our result is selective for decisions involving losses, but not gains, which mitigates the suggestion that the effect seen is caused by changes in wakefulness affecting risk taking overall. In fact sleep deprivation has been shown to increase risk taking in gains and decrease in losses^61^. Future work might usefully examine how chronotypes relate to a modulation of diurnal changes in loss sensitivity. Finally, it is of interest that profound changes are reported in resting-state brain network connectivity profiles over the course of the day^36^. Integrating the latter approach in conjunction with measures of decision making could help to identify the specific networks that underlie diurnal changes in loss sensitivity.

We observed an effect of time of day on risk taking in mixed trials in the between subjects’ analysis in female but not male participants. We did not observe a difference in mixed trials for the within-subjects analysis, but this may be due to the smaller sample size, and the fact that we presented participants with fewer mixed trials (eight) than loss or gain trials (11 of each). Sex differences in risk taking have been observed in a variety of effects (e.g., under stress^62,63^, sensitivity to winning and losing^64^ and real life risky behaviors such as drug use and dangerous driving^65^) but little is known about how these may interact with the circadian rhythms of neuromodulators. Speculatively, one possible reason is human sex hormones interacting with different phases of circadian rhythms. Estrogen has been associated with shortened circadian periods in rodents (i.e., more free running in daylight hours^66^) and has been suggested to shorten the circadian period in humans^67^. Increased estrogen during ovulation has also been associated with reduced loss aversion in mixed trials in humans^68^. Future work should focus on more purposefully sampling to further explore gender differences in circadian rhythms that we were not able to investigate in our within-subjects analyses.

Our results demonstrate that diurnal patterns in cognition and behavior can be remotely assessed with smartphones. This also provides a non-invasive method to observe circadian rhythm disruption, a key factor in many mental health disorders^69,70^. The causal direction of circadian rhythm disruption and psychiatric disorders is unknown^71^. Further work exploring the interaction of hormones and circadian cycles may also shed light on the increased prevalence of seasonal affective disorder in women which has been associated with disrupted circadian rhythms^72,73^. The advancement of smartphone platform and online testing provides a powerful methodological framework where frequent sampling of behavior in the morning and evening can provide an efficient way to measure circadian rhythm disruption in relation to mental health disorders^40^. Gamification can also make a task more engaging, which may be particularly valuable in the study of adolescent behavior when mental health problems peak^74^, with potential for early interventions. Future work could investigate if this effect becomes stronger when real money is at stake, as for example shown in the comparison of risk taking for real and hypothetical rewards^75^.

Many factors contribute to human risk taking and it would be surprising for gradual circadian changes to elicit large effects on risk taking. The robust effects on loss sensitivity that we observe could have significant implications at the societal level. International stock markets are simultaneously at different positions in their diurnal patterns (i.e., the New York Stock Exchange (NYSE) opens in the morning around when the Tokyo exchange ends trading for the day). Individuals making decisions about purchasing or selling stock from an international exchange may exhibit differences in loss sensitivity to individuals making purchases in local time zones. Given NYSE opening hours, investors in California may make decisions about NYSE-listed stocks earlier in their day on average than investors in Berlin. Time of day is known to be relevant to investor behavior, for example traders operating at circadian-mismatched times of day have been shown to use riskier strategies resulting in lower earnings when competing with traders at circadian-matched times^76^. Our finding of lower sensitivity to potential losses late in the day draws attention to one potential factor that may contribute to this effect. Policy changes that allow for more voting in the early morning, or late evening, could also have profound implications particularly if voters view candidates in a loss frame (i.e., they dislike both candidates) where we have shown that risk taking increases throughout the day. Clinicians often make important medical decisions late at night, but it is unknown whether those decisions are different from the ones they would make in the morning in important ways dependent on the decision framing. Understanding how diurnal biases in risk taking affect behavior at individual and population level is useful for policy makers and might shape decision making across a wide range of domains.

## Supplementary Information

Supplementary Information.

## Data Availability

The datasets analysed during the current study are available in a Dryad repository, https://doi.org/10.5061/dryad.prr4xgxkk*.*

## Code availability

Code to generate the figures is avaliable at https://github.com/RachelBedder/RiskTaking_ScientificReports.
