# Supplementary material for: Risk taking for potential losses but not gains increases with time of day
Source: Sci Rep. 2023 Apr 4;13:5534. doi: 10.1038/s41598-023-31738-x (PMC10073197; doi:10.1038/s41598-023-31738-x)
Supplement: Supplementary file 1 — Supplementary Information. [file 41598_2023_31738_MOESM1_ESM.docx]

**SUPPLEMENTARY INFORMATION**

**Supplementary Methods**

**Trial Design**

The initial app release (March 6, 2013 until July 16, 2013) featured a ratio design that generated the values for the risky choices as fixed multipliers of the safe choice for gain and loss trials). The gain trials had 4 possible safe amounts (30, 35, 45, 55) and possible 15 multipliers on those amounts to generate potential gamble gains (1.64, 1.7, 1.76, 1.82, 1.88, 1.94, 2, 2.06, 2.12, 2.18, 2.26, 2.4, 2.7, 3.2, 4). For every game 11 safe amounts and ratios were randomly selected to generate the gain trials. The loss trial set was identical to the gain trial set except with negative values; 11 new safe amounts and ratios were sampled for loss trials. Mixed trials had 3 prospective gains (40, 44, 75) with 10 prospective losses generated from multipliers on those amounts (0.2, 0.34, 0.6, 0.54, 0.77, 0.89, 1, 1.1, 1.35, 2). Eight trials with random prospect gains and loss multipliers were randomly selected for each game. In the ratio design, potential outcomes are correlated by design. Later users played an ‘uncorrelated’ design where the values of the safe and risky choices were all randomly sampled from a set of amounts within each task domain. Gain trials had 3 safe amounts (35, 45, 55) that were each randomly paired with one of 15 possible gamble gain amounts (59, 66, 72, 79, 85, 92, 98, 105, 111, 118, 124, 131, 137, 144, 150) to generate 11 gain trials. The loss trial set was identical to the gain trial set except with negative values; 11 new safe amounts and gamble amounts were sampled for loss trials. Mixed trials had 3 prospective gains (40, 44, 75) randomly paired with 10 prospective losses (-10, -19, -28, -37, -46, -54, -63, -72, -81, -90), which were randomly selected to generate 8 mixed trials.

**SUPPLEMENTARY RESULTS**

**Supplementary Table 1. Descriptive Statistics for Model Free and Model Based Measures**

|  |  |  | Proportion of risky options chosen | | | Proportion of choices with highest expected value | | | Points Scored | | | Dual Alpha Model | | | | Single Alpha Model | | |
| --- | --- | --- | --- | --- | --- | --- | --- | --- | --- | --- | --- | --- | --- | --- | --- | --- | --- | --- |
|  |  |  | Gain | Loss | Mixed | Gain | Loss | Mixed | Gain | Loss | Mixed | α_gain_ | α_loss_ | log(λ) | μ | α | log(λ) | μ |
| All |  | **Mean**  (S.D)  Median | **0.70** (0.26) 0.73 | **0.54** (0.30) 0.55 | **0.66**  (0.26) 0.63 | **0.65**  (.19)  0.64 | **0.59** (0.20)  0.64 | **0.65** (0.19) 0.63 | **556**  (164) 543 | **-499** (131) -490 | **35**  (129) 36 | **1.02** (0.30) 1.11 | **0.79** (0.34) 0.79 | **0.63** (0.93) 0.81 | **0.74** (2.76)  0.089 | **0.78** (0.33)  0.79 | **0.58**  (0.97) 0.76 | **0.38** (2.07)  .095 |
|  | 6am | **Mean**  (S.D) | **0.70**  (0.26) | **0.52** (0.29) | **0.65**  (0.26) | **0.65** (0.19) | **0.60**  (0.21) | **0.66**  (0.19) | **556** (162) | **-496** (129) | **36**  (127) | **1.03**  (0.30) | **0.81**  (0.34) | **0.61**  (0.93) | **0.76**  (2.91) | **0.80**  (0.33) | **0.55**  (0.96) | **0.41**  (2.20) |
|  | 12pm | **Mean**  (S.D) | **0.70** (0.26) | **0.53** (0.29) | **0.66**  (0.26) | **0.65** (0.18) | **0.60** (0.20) | **0.65**  (0.19) | **556** (167) | **-500**  (129) | **36**  (127) | **1.02**  (0.30) | **0.80**  (0.34) | **0.62**  (0.93) | **0.80**  (2.91) | **0.79**  (0.32) | **0.57**  (0.97) | **0.41**  (2.14) |
|  | 6pm | **Mean**  (S.D) | **0.70** (0.26) | **0.54** (0.30) | **0.67** (0.26) | **0.64**  (0.18) | **0.59**  (0.20) | **0.64**  (0.19) | **555**  (164) | **-499**  (132) | **35**  (130) | **1.02** (0.30) | **0.78**  (0.34) | **0.63**  (0.93) | **0.69**  (2.64) | **0.77**  (0.33) | **0.59**  (0.97) | **0.35**  (1.93) |
|  | 12am | **Mean**  (S.D) | **0.70** (0.26) | **0.56** (0.30) | **0.67** (0.26) | **0.65**  (0.19) | **0.58**  (0.21) | **0.64**  (0.19) | **555**  (165) | **-502**  (133) | **36**  (131) | **1.02**  (0.30) | **0.76**  (0.34) | **0.66**  (0.93) | **0.74**  (2.67) | **0.76**  (0.33) | **0.63**  (0.97) | **0.40**  (2.15) |

**Supplementary Table 1** (continued).

|  |  |  | Proportion of risky options chosen | | | Proportion of choices with highest expected value | | | Points Scored | | | Dual Alpha Model | | | | Single Alpha Model | | |
| --- | --- | --- | --- | --- | --- | --- | --- | --- | --- | --- | --- | --- | --- | --- | --- | --- | --- | --- |
|  |  |  | Gain | Loss | Mixed | Gain | Loss | Mixed | Gain | Loss | Mixed | α_gain_ | α_loss_ | log(λ) | μ | α | log(λ) | μ |
| Female |  | **Mean**  (S.D)  Median | **0.71**  (0.27) 0.82 | **0.54**  (0.30) 0.55 | **0.68** (0.26) 0.75 | **0.61**  (0.18)  0.64 | **0.56** (0.19) 0.55 | **0.64** (0.18) 0.63 | **548** (164) 533 | **-501** (131) -493 | **32**  (132)  31 | **1.02**  (0.32) 1.14 | **0.77** (0.36) 0.74 | **0.62** (0.95) 0.82 | **0.55** (2.24) 0.078 | **0.76** (0.34) 0.74 | **0.59** (1.00) 0.85 | **0.16** (1.05) 0.087 |
|  | 6am | **Mean** (S.D) | **0.71**  (0.27) | **0.52** (0.30) | **0.66**  (0.26) | **0.62**  (0.18) | **0.58**  (0.19) | **0.64**  (0.18) | **549**  (166) | **-499**  (130) | **31**  (131) | **1.02**  (0.32) | **0.80**  (0.35) | **0.61**  (0.94) | **0.61**  (2.52) | **0.79**  (0.34) | **0.55**  (0.99) | **0.23**  (1.48) |
|  | 12pm | **Mean** (S.D) | **0.70**  (0.28) | **0.54** (0.31) | **0.66**  (0.27) | **0.62**  (0.17) | **0.57**  (0.19) | **0.62**  (0.18) | **544**  (164) | **-502**  (130) | **32**  (129) | **1.01**  (0.32) | **0.77**  (0.35) | **0.61**  (0.95) | **0.61**  (2.35) | **0.77**  (0.34) | **0.58**  (1.00) | **0.20**  (1.24) |
|  | 6pm | **Mean** (S.D) | **0.71**  (0.27) | **0.55**  (0.30) | **0.69**  (0.26) | **0.61**  (0.17) | **0.56**  (0.19) | **0.62**  (0.18) | **550**  (163) | **-500** (131) | **33**  (132) | **1.02**  (0.32) | **0.76**  (0.36) | **0.62**  (0.95) | **0.50**  (2.10) | **0.75**  (0.34) | **0.59**  (1.00) | **0.15**  (0.75) |
|  | 12am | **Mean** (S.D) | **0.71**  (0.27) | **0.57**  (0.30) | **0.70**  (0.26) | **0.60**  (0.18) | **0.55**  (0.20) | **0.62**  (0.18) | **544**  (163) | **-503**  (136) | **34**  (138) | **1.02**  (0.32) | **0.76**  (0.36) | **0.64**  (0.95) | **0.55**  (2.14) | **0.74**  (0.34) | **0.64**  (1.00) | **0.16**  (0.85) |
| Male |  | **Mean**  (S.D)  Median | **0.70** (0.25) 0.73 | **0.53** (0.28) 0.55 | **0.65** (0.26) 0.63 | **0.68** (0.19) 0.73 | **0.62** (0.21) 0.64 | **0.67** (0.20) 0.63 | **563** (164)  552 | **-497** (130)  -486 | **38**  (126) 40 | **1.03** (0.29) 1.09 | **0.81**  (0.32) 0.82 | **0.63** (0.92) 0.79 | **0.92** (3.16) 0.100 | **0.80** (0.31) 0.82 | **0.58** (0.94) 0.71 | **0.58** (2.67) 0.10 |
|  | 6am | **Mean**  (S.D) | **0.70**  (0.25) | **0.52**  (0.29) | **0.64**  (0.25) | **0.68**  (0.19) | **0.63**  (0.22) | **0.67**  (0.19) | **562**  (158) | **-493**  (129) | **40**  (124) | **1.03**  (0.28) | **0.82**  (0.32) | **0.62**  (0.92) | **0.88**  (3.09) | **0.82**  (0.32) | **0.54**  (0.94) | **0.57**  (2.65) |
|  | 12pm | **Mean**  (S.D) | **0.70**  (0.25) | **0.52**  (0.28) | **0.65**  (0.26) | **0.68**  (0.19) | **0.63**  (0.21) | **0.67**  (0.20) | **566**  (168) | **-497**  (128) | **39**  (125) | **1.03**  (0.28) | **0.82**  (0.32) | **0.63**  (0.92) | **0.90**  (3.29) | **0.81**  (0.31) | **0.55**  (0.95) | **0.58**  (2.64) |
|  | 6pm | **Mean**  (S.D) | **0.69**  (0.25) | **0.53**  (0.29) | **0.65**  (0.26) | **0.67**  (0.19) | **0.61**  (0.21) | **0.66**  (0.19) | **562**  (164) | **-498**  (133) | **37**  (127) | **1.02**  (0.29) | **0.80**  (0.32) | **0.64**  (0.92) | **0.90**  (3.13) | **0.79**  (0.32) | **0.60**  (0.94) | **0.57**  (2.69) |
|  | 12pm | **Mean**  (S.D) | **0.70**  (0.25) | **0.55**  (0.29) | **0.65**  (0.26) | **0.68**  (0.19) | **0.61**  (0.22) | **0.66**  (0.20) | **564**  (165) | **-500**  (131) | **39**  (125) | **1.03**  (0.29) | **0.79** (0.32) | **0.67**  (0.91) | **0.91**  (3.06) | **0.78**  (0.32) | **0.62**  (0.95) | **0.62**  (2.84) |

**Supplementary Table 2. Effect Sizes for Time of Day on Choosing Highest Expected Value and Total Points Scored for Male and Female Participant Splits.**

|  | Proportion of choices with highest expected value | | | | | | Points Scored | | | | | |
| --- | --- | --- | --- | --- | --- | --- | --- | --- | --- | --- | --- | --- |
| Data | Gain | | Loss | | Mixed | | Gain | | Loss | | Mixed | |
|  | Effect size | p value  BF_01_ | Effect size | p value  BF_01_ | Effect size | p value  BF_01_ | Effect size | p value  BF_01_ | Effect size | p value  BF_01_ | Effect size | p value  BF_01_ |
| All | -0.028 | < 0.0001 0.003 | -0.041 | <0.0001 2.15 × 10^-8^ | -0.028 | <0.0001  0.005 | -0.0040 | 0.515 105.01 | -0.011 | 0.082 29.64 | -0.0014 | 0.82  127.00 |
| Female | -0.024 | 0.0084  2.43 | -0.042 | <0.0001  7.47 × 10^-4^ | -0.024 | 0.0049  2.06 | -0.0023 | 0.791  88.17 | -0.007 | 0.42  65.96 | 0.0064 | 0.47  69.61 |
| Male | -0.018 | 0.374  10.65 | - 0.029 | 0.0009  0.28 | -0.022 | 0.0109  3.39 | -0.0015 | 0.859  91.89 | - 0.013 | 0.15 31.85 | -0.00068 | 0.44  67.77 |

Effect sizes (Pearson’s r) for each all participants and split for female and male participant demographic groups (total N= 26,270). All Bayes Factor tests test for evidence for the null hypothesis (BF_01_). BF_01_ of > 1 indicate support for the null hypothesis.

**Supplementary Table 3. Effect Sizes for Time of Day on Single Alpha Model Parameters**

|  | α | | log(λ) | | μ | |
| --- | --- | --- | --- | --- | --- | --- |
| Data | Effect size | p value  BF_01_ | Effect size | p value  BF_01_ | Effect size | p value  BF_01_ |
| All | -0.040 | <0.0001  5.20 × 10^-8^ | 0.029 | <0.0001  0.001 | -0.0079 | 0.19  56.77 |
| Female | -0.041 | <0.0001  0.002 | 0.018 | 0.036  10.62 | -0.032 | 0.0003  0.12 |
| Male | -0.034 | <0.0001  0.031 | 0.018 | <0.0001  0.093 | 0.00064 | 0.449  70.49 |
| Within-Subjects Subsample | -0.053 | 0.0069  1.11 | 0.045 | 0.020  2.99 | -0.019 | 0.34  26.05 |

Effect sizes (Pearson’s r) for each all participants and split for female and male participant demographic groups (total N= 26,270) and the within-subjects subsample (N = 2,599). Bayes Factor (BF_01_) tests for evidence for the null hypothesis are included in all instances where the permutated p value > 0.01. BF_01_ of > 1 indicate support for the null hypothesis.
